# 

**Published:** 1893-08

**Authors:** 


### CONTENTS:

Article I.—Three New Remedial Preparations. By Dr. W. E. Walker. - - - - - - - 145

" II.—Discoloration of Gold Fillings. - - - - - 148

" III.—Things Practical in Dental Practice. By J. G. Templeton, D. D. S. - - - - - - 151

" IV.—Disease in the Antrum. By J. B. Van Fossen, D. D. S. 154

" V.—The Great Ordeal. By Dr. W. S. Elliott. - - 156

" VI.—No American Dental Diplomas Recognized by the General Medical Council of Great Britain. - - 159

### COMMUNICATED.

World's Columbian Dental Congress. - - - - - - 175

Resolutions Adopted by the Chicago Dental Society on the Death of Dr. W. W. Allport. - - - - - - 181

Twenty-fourth Annual Meeting of the California State Dental Society. - - - - - - - - 182

Annual Meeting of the National Association of Dental Faculties. - 182

### EDITORIAL.

The Progress of Surgery. - - - - - - - - . 183

Oral and Facial Surgery. - - - - - - - - - 184

### MONTHLY SUMMARY.

The Use of Anæsthetics in Extracting Teeth. - - - - - 187

Cements as Root Fillings. - - - - - - - - - 189

In Using Cocaine. - - - - - - - - - - 190

Philosophers and Cranks. - - - - - - - - 190

New Method of Adjusting Logan Crowns. - - - - - 191

Presence of Mind in Applying an Antidote. - - - - - 191

Dr. Wm. Crenshaw. - - - - - - - - - - 192

